# Methylphenidate enhances neural stem cell differentiation

**DOI:** 10.1186/2049-9256-1-5

**Published:** 2013-04-23

**Authors:** Jasmin Bartl, Takatoshi Mori, Peter Riederer, Hiroki Ozawa, Edna Grünblatt

**Affiliations:** 1 Hospital of Child and Adolescent Psychiatry, University of Zurich, Winterthurerstr. 180, Room L84/86, 8057 Zurich, Switzerland; 2 Division of Neuropsychiatry, Nagasaki University Graduate School of Biomedical Sciences, Nagasaki, Japan; 3 Department of Psychiatry, Psychosomatics and Psychotherapy, University Hospital of Wuerzburg, Wuerzburg, Germany; 4 Neuroscience Center Zurich, University of Zurich and ETH Zurich, Kragujevac, Switzerland

**Keywords:** Methylphenidate, Neural stem cells, Neuronal maturation, Cell proliferation

## Abstract

**Background:**

The psychostimulant methylphenidate (MPH) is the first choice of drug treatment in Attention-Deficit/Hyperactivity Disorder (ADHD). Since therapy often begins at a time when the brain is still developing and the long-term consequences of MPH are still not fully clarified, we examined the influences of an acute treatment with MPH on the differentiation and proliferation of murine neural stem cells (mNSC).

**Findings and conclusion:**

We found that MPH enhanced neuronal differentiation and inhibited neural proliferation.

## Findings

### Background

ADHD is one of the most frequent psychiatric disorders in children and adolescents, with up to 5% affected worldwide and similar prevalence rates throughout different cultural settings [1, 2]. It is characterized by developmentally inappropriate levels of inattention, hyperactivity, and/or impulsivity. These core symptoms lead to impairment in home, school, and peer contexts. Stimulants, particularly MPH, are part of the first-line treatment therapies for ADHD [3] and in recent years, the number of prescriptions has increased nearly tenfold [4]. Mechanistically, MPH is a high-affinity inhibitor of the dopamine transporter and a middle-affinity inhibitor to the norepinephrine transporter [5]. However, given the extent of the prescribed use of MPH [6] exposure to MPH during the early stages of brain development raises some concern for public health due to possible adverse long-term effects such as neurogenesis, neuronal development, or receptor density. It is not clear whether such changes occur; if they do, whether they are related to medication or to the condition itself; if they are caused by medication, whether they have functional significance; and whether any changes are helpful or harmful to mental development. Still, the full mechanism of action of MPH has not yet been elucidated. A recent study by Lee and colleagues (2012) investigated *in vivo* the effect of chronically treatment of MPH on cell proliferation and neuronal differentiation in adolescent mice brain tissue [7]. They could demonstrate that 10 mg/kg MPH treatment for 28 days enhances cell proliferation as well as neuroblast differentiation in contrast to Lagace and colleagues (2006), who detected an inhibition of survival of adult-generated neurons in the temporal hippocampus of adolescent rats after 16 days treatment with 2 mg/kg of MPH [8]. In concern to the contrasting results and experimental designs of *in vivo* studies, we investigated the *in vitro* effect of an acute treatment with MPH using murine neural stem cells (mNSC) originating from hippocampal tissue of embryonic mice E15. NSC are neurosphere-forming cells and can serve as a model for basic neurodevelopmental processes as well as a potential source of neurodegenerative disease [9]. They are clonogenic, self-renewing, and multipotent cells with plasticity to proliferate and to differentiate into all cell types of the central nervous system (CNS) such as glia/astrocytes, oligodendrocytes, and neurons [10]. Such a model enables the study of neuronal development, differentiation, and neuronal cell death mechanisms *in vitro*.

## Material and methods

### Murine neural stem cell (mNSC) sphere culturing

mNSCs were derived from the hippocampus tissue of albino mouse (Charles River, Japan) embryos on embryonic day 15. Cells were cultured in a medial hormone mix (MHM) with 10 μg/ml epidermal (EGF; Sigma aldrich, Japan, Cat-No: SRP3196) and 10 μg/ml basic fibroblast growth factors (bFGF; Sigma aldrich, Japan, Cat-No: F5392) for six days at 37°C and 5% CO2. After six days, the grown NSC spheres were harvested and prepared for differentiation and/or proliferation studies (for methods, see below).

### Differentiation study

mNSC spheres were collected and plated on a 30 μg/ml poly-l-lysine (Sigma aldrich, Japan, Cat-No: P1274) and 20 μg/ml laminine (Invitrogen, Japan, Cat-No: 23017–015) coated 8-well glass cover-slip and incubated for another 4 days. Each well had a final concentration of 22.8x104 cells/ml cultured in MHM containing 1% fetal bovine serum (FBS) and without EGF and bFGF. The cells were treated directly after seeding to the cover-slip using different concentration (0 nM, 1 nM, 10 nM, 100 nM) of methylphenidate (MPH; Sigma aldrich, Japan, Cat-No: M2892). On the last day, cells were fixed with 4% paraformaldehyde (Fluka, Japan, Cat-No: 76240) for 20 minutes at room temperature (RT) and stained with different antibodies (see “Immunocytochemistry”).

### BrdU incorporation

The proliferation of NSC was identified by in vitro labeling with the thymidine analog 5-bromo-2-desoxyuridine (BrdU; Wako, Japan, Cat-No: 023–15563). NSC spheres were collected and plated on coated 8-well glass cover-slip and incubated for 24 h. Each well had a final concentration of 22.8x104 cells/ml cultured in MHM, containing EGF and bFGF and without FBS. The cells were treated with different concentration of MPH (0 nM, 1 nM, 10 nM, 100 nM). After 24 h, BrdU was added to each well to reach a final concentration of 10 μM and incubated for 4 h at RT. Before BrdU immunostaining, DNA was denatured and the nucleus-membrane was broken by treating cells with 2M HCL for 35 min at RT; afterward, cells were rinsed with 1% phosphate buffer saline (PBS), followed by treatment with sodium borate (pH=8.5) for 10 min at RT for the neutralization of HCL. The prepared cells were stained against BrdU (see “Immunocytochemistry”).

### Immunocytochemistry

Fixed and prepared cells were stained for differentiation studies with rabbit monoclonal antibody against glial fibrillary acidic protein (GFAP; 1:200, Sigma aldrich, USA, Cat-No: C4546) and/or with mouse monoclonal antibody against β-tubulin III (Tuj 1; 1:300, Sigma aldrich, USA, Cat-No: T3952) overnight at 4°C. For proliferation studies, cells were stained with rat monoclonal antibody against BrdU (1:100, Accurate Chemical & Scientific, Japan, Cat-No: OBT0030) overnight at 4°C. In both studies, fixed cells were also stained with Hoechst 33258 (1:100; Invitrogen, Cat-No: H3569) for 15 min at RT to visualize the cell nuclei. After overnight incubation, the primary antibodies were visualized with secondary antibodies against rabbit, mouse, or rat conjugated to the following fluorochromes: Alexa Fluor® -488 and Alexa Fluor® -555 (Life Technologies, Japan).

### Statistical analysis

The stainings were analyzed for the cell count of astrocytes, immature neurons and proliferated cells in comparison to the total number of cells using the Mann–Whitney (U-Rang) Test in the StatView software program (Stat View 5.0. software, SAS Institute Inc. Cary, NC, USA). A p-value < 0.05 was set as significant. There were at least five independent experiments, and four wells per slide were analyzed. In each case, the total amount of Hoechst stained cells/well were manually counted, and afterward, either BrdU positive, GFAP positive, or Tuj 1 positive cells were counted and the percentage of positive cells to the total amount was calculated. In the proliferation and differentiation studies a comparison of the treated mNSC to the control mNSC (MPH untreated) was done.

## Results

**Figure 1:**
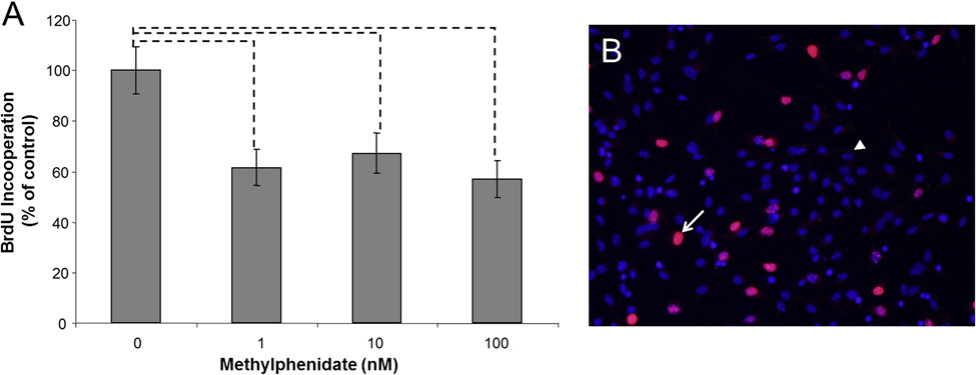
**Murine neural stem cell (mNSC) differentiation into immature neurons. A**) mNSCs were treated with different concentration (0 nM, 1 nM, 10 nM, 100 nM) of methylphenidate (MPH). The percentage (% control) of developed neurons was determined 4 days after treatment with MPH. The amount of immature neurons was analyzed by counting the neuron-specific class III beta-tubulin (Tuj 1) positive cells in comparison to the total number of cells by using the Mann–Whitney (U-Rang) Test; --- =p <0.05; n= 28; seven independent experiments and four wells/slide of each concentration were evaluated. **B**) An example of an immunocytochemistry staining of Tuj 1 in a control sample (no MPH treatment). The white arrow points to a Tuj 1 positive cell (green) and the white arrow points to a cell nucleus staining with Hoechst (blue); 40x magnification.

**Figure 2:**
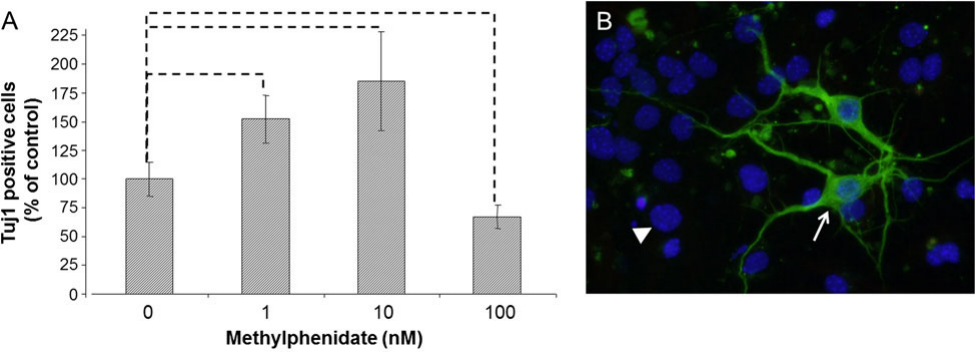
**Murine neural stem cell (mNSC) proliferation. A**) mNSCs were treated with different concentration (0 nM, 1 nM, 10 nM, 100 nM) of methylphenidate (MPH). The percentage (% of control) of proliferated cells was determined 28 h after induction of neuronal proliferation and MPH treatment. The amount of proliferating cells was analyzed by counting the Bromodeoxyurdine (BrdU) positive cells in comparison to the total number of cells using the Mann–Whitney (U-Rang) Test; --- =p <0.05; n= 20; five independent experiments and four wells/slide of each concentration were evaluated. **B**) An example of an immunocytochemistry staining of BrdU in a control sample (no MPH treatment). The white arrow points to a BrdU positive cell (red) and the white arrow points to a BrdU negative cell (blue); 10x magnification.

**Figure 3:**
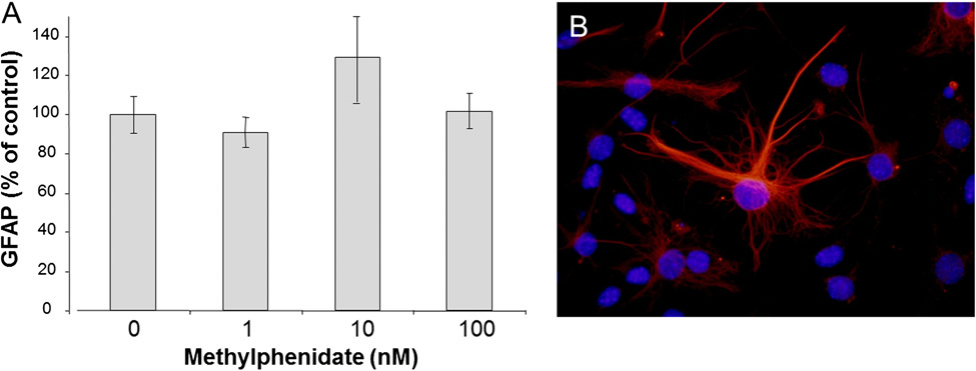
**Murine neural stem cell (mNSC) differentiation into astrocytes. A**) mNSCs were treated with different concentration (0 nM, 1 nM, 10 nM, 100 nM) of methylphenidate (MPH). The percentage (% control) of developed neurons was determined 4 days after treatment with MPH. The amount of astrocytes was analyzed by counting the glial fibrillary acidic protein (GFAP) positive cells in comparison to the total number of cells using the Mann–Whitney (U-Rang) Test; --- =p <0.05; n= 28; seven independent experiments and four wells/slide of each concentration were evaluated. **B**) An example of an immunocytochemistry staining of GFAP in a control sample (no MPH treatment). GFAP positive cell (red) and cell nucleus staining with Hoechst (blue); 40x magnification.

After 1 nM MPH treatment, the neuronal differentiation was significantly enhanced, and 53% more immature neurons could be detected compared to untreated mNSCs (Figure 1). After 10 nM of MPH, the enhancement of neuronal differentiation was over 80%, but the highest dose of MPH (100 nM) showed a reduction of 39% (Figure 1). In contrast, all tested concentration (1-100 nM) of MPH inhibited the proliferation of mNSC (Figure 2). For neuronal maturation, it is important that the neural stem cell stops proliferating and starts the outgrowth of neuritis to develop into full neurons, which seems to be enhanced by the concentration of 1 nM and 10 nM of used MPH. In contrasts, MPH treatment at the given dose range did not significant affect the development of astrocytes in comparison to untreated mNSC (Figure 3).

## Availability of supporting data

### Discussion

In this work, we presented the effects of low doses (1-100 nM) MPH treatment at mNSC proliferation and neuronal maturation. In 2010 we could already demonstrate, that especially low doses of MPH have the strongest impact on gene expression and cell proliferation in neuronal cell line culture [11]. Our results suggest that MPH seems to support neuronal maturation in a specific range and enhances the neuronal outcome. This hypothesis fits the fact that children with ADHD have atypical or typical but delayed maturation of the prefrontal cortex [12]. Structural imaging in ADHD provides evidence for a global maturational delay based on reduced gray and white matter volume and cortical thickness in ADHD relative to controls through childhood and adolescence [13, 14]. MPH seems to balance this retardation of neuronal development, but the mechanism of action is still not known and needs further investigation [15]. However, few studies have addressed the structural correlates of psychostimulant treatment. Castellanos et al. (2002) found that prior treatment with psychostimulants in children with ADHD was associated at study entry with greater white matter lobar volumes relative to stimulant-naive children with ADHD and volumes that lie closer to the range of their typically developing counterparts, suggesting a neuroprotective effect [16]. An independent study of 30 children with ADHD examining the regions implicated in the pathogenesis of the disorder, similarly, found that treatment with psychostimulants was associated with a more normative volume of the caudate and anterior cingulate cortex [17]. Additionally, the group of Antonello Bonci (2010) recently showed that MPH can influence neuronal plasticity in the amygdale and, thus, can improve learning performance [18]. Within the inherent limitations of a preliminary study, we find great influences of MPH on neuronal maturation, which may prove activity-dependent neuronal plasticity. However, one has to take into account that this *in vitro* experiment did not investigate any MPH influences on neurogenesis, which can be another possible mechanism of action of MPH.

## Abbreviations

ADHD: Attention-Deficit/Hyperactivity Disorder
bFGF: basic fibroblast growth factor
BrdU: 5-bromo-2-desoxyuridine
CNS: Central nervous system
CO2: Carbon dioxid
EGF: Epidermal growth factor
FBS: Fetal bovine serum
GFAP: Glial fibrillary acicid protein
HCL: Hydrochloric acid
MHM: Medial hormone mix
mNSC: Murine neural stem cell
MPH: Methylphenidate
nM: Nano mol
RT: Room temperature
Tuj1: Beta tubulin III.
