# Correction: Methylphenidate enhances neural stem cell differentiation

**DOI:** 10.1186/2049-9256-1-10

**Published:** 2013-06-10

**Authors:** Jasmin Bartl, Takatoshi Mori, Peter Riederer, Hiroki Ozawa, Edna Grünblatt

**Affiliations:** Hospital of Child and Adolescent Psychiatry, University of Zurich, Winterthurerstr. 180, Room L84/86, Zurich, 8057 Switzerland; Division of Neuropsychiatry, Nagasaki University Graduate School of Biomedical Sciences, Nagasaki, Japan; Department of Psychiatry, Psychosomatics and Psychotherapy, University Hospital of Wuerzburg, Wuerzburg, Germany; Neuroscience Center Zurich, University of Zurich and ETH Zurich, Zurich, Switzerland

## Correction

After the publication of this work [1], we noticed an error whereby the images of Figure 1 and Figure 2 are interchanged and therefore do not correspond to their legends. The image of Figure 1 belongs to Figure 2 and *vice versa*. The corrected figures are given below.

**Figure 1:**
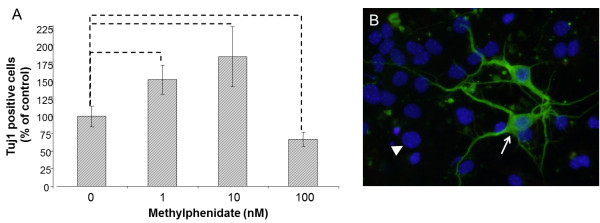
**Murine neural stem cell (mNSC) differentiation into immature neurons. A**) mNSCs were treated with different concentration (0nM, 1nM, 10nM, 100nM) of methylphenidate (MPH). The percentage (% control) of developed neurons was determined 4 days after treatment with MPH. The amount of immature neurons was analyzed by counting the neuron-specific class III beta-tubulin (Tuj 1) positive cells in comparison to the total number of cells by using the Mann–Whitney (U-Rang) Test; --- = p <0.05; n = 28; seven independent experiments and four wells/slide of each concentration were evaluated. **B**) An example of an immunocytochemistry staining of Tuj 1 in a control sample (no MPH treatment). The white arrow points to a Tuj 1 positive cell (green) and the white arrow points to a cell nucleus staining with Hoechst (blue); 40x magnification.

**Figure 2:**
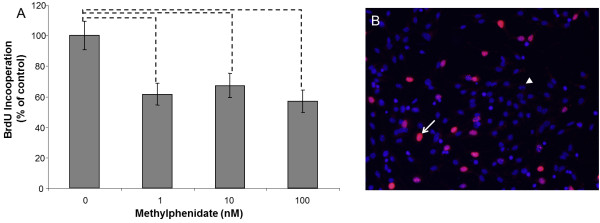
**Murine neural stem cell (mNSC) proliferation. A**) mNSCs were treated with different concentration (0nM, 1nM, 10nM, 100nM) of methylphenidate (MPH). The percentage (% of control) of proliferated cells was determined 28 h after induction of neuronal proliferation and MPH treatment. The amount of proliferating cells was analyzed by counting the Bromodeoxyurdine (BrdU) positive cells in comparison to the total number of cells using the Mann–Whitney (U-Rang) Test; --- = p <0.05; n = 20; five independent experiments and four wells/slide of each concentration were evaluated. **B**) An example of an immunocytochemistry staining of BrdU in a control sample (no MPH treatment). The white arrow points to a BrdU positive cell (red) and the white arrow points to a BrdU negative cell (blue); 10x magnification.
